# An Inhalable Powder Formulation Based on Micro- and Nanoparticles Containing 5-Fluorouracil for the Treatment of Metastatic Melanoma

**DOI:** 10.3390/nano8020075

**Published:** 2018-01-30

**Authors:** Kelly Cristine Zatta, Luiza A. Frank, Luciano Antonio Reolon, Lucas Amaral-Machado, Eryvaldo S. T. Egito, Maria Palmira Daflon Gremião, Adriana Raffin Pohlmann, Silvia S. Guterres

**Affiliations:** 1 Programa de Pós-Graduação em Nanotecnologia, Universidade Federal do Rio Grande do Sul (UFRGS), Porto Alegre RS 90610-000, Brazil; kellycriz@hotmail.com (K.C.Z.); adriana.pohlmann@ufrgs.br (A.R.P.); 2 Programa de Pós-Graduação em Ciências Farmacêuticas, Universidade Federal do Rio Grande do Sul (UFRGS), Porto Alegre RS 90610-000, Brazil; luiza.frank@ufrgs.br; 3 Escola de Ciências da Saúde, Centro Universitário Ritter dos Reis–UniRitter, Porto Alegre RS 90840-440, Brazil; luciano_reolon@uniritter.edu.br; 4 Programa de Graduação em Ciências da Saúde, Universidade Federal do Rio Grande do Norte (UFRN), Natal RN 59012-570, Brazil; machado.lucasam@gmail.com (L.A.-M.); socratesegito@gmail.com (E.S.T.E.); 5 Programa de Pós-Graduação em Ciências Farmacêuticas, Universidade Estadual de São Paulo (UNESP), Araraquara SP 14801-903, Brazil; pgremiao@fcfar.unesp.br; 6 Departamento de Química Orgânica, Instituto de Química, Universidade Federal do Rio Grande do Sul (UFRGS), Porto Alegre RS 90650-001, Brazil

**Keywords:** 5-fluorouracil, biopolymers, metastatic melanoma, pulmonary delivery, microparticles, nanoparticles, cytotoxicity

## Abstract

Melanoma is the most aggressive and lethal type of skin cancer, with a poor prognosis because of the potential for metastatic spread. The aim was to develop innovative powder formulations for the treatment of metastatic melanoma based on micro- and nanocarriers containing 5-fluorouracil (5FU) for pulmonary administration, aiming at local and systemic action. Therefore, two innovative inhalable powder formulations were produced by spray-drying using chondroitin sulfate as a structuring polymer: (a) 5FU nanoparticles obtained by piezoelectric atomization (5FU-NS) and (b) 5FU microparticles of the mucoadhesive agent Methocel™ F4M for sustained release produced by conventional spray drying (5FU-MS). The physicochemical and aerodynamic were evaluated in vitro for both systems, proving to be attractive for pulmonary delivery. The theoretical aerodynamic diameters obtained were 0.322 ± 0.07 µm (5FU-NS) and 1.138 ± 0.54 µm (5FU-MS). The fraction of respirable particles (FR%) were 76.84 ± 0.07% (5FU-NS) and 55.01 ± 2.91% (5FU-MS). The in vitro mucoadhesive properties exhibited significant adhesion efficiency in the presence of Methocel™ F4M. 5FU-MS and 5FU-NS were tested for their cytotoxic action on melanoma cancer cells (A2058 and A375) and both showed a cytotoxic effect similar to 5FU pure at concentrations of 4.3 and 1.7-fold lower, respectively.

## 1. Introduction

Cutaneous melanoma is the most aggressive skin cancer, which starts as an injury restricted to the most superficial layers of the skin [1]. In its early stages, the principal treatment is based on surgical removal, which offers good chances of patient survival [2]. However, in advanced stages, melanoma exhibits rapid growth, high potential of metastatic spread and high lethality [3]. In stage IV, which is referred to as metastatic melanoma (MM), cancer cells are widespread in the body, reaching remote areas such as the lungs, liver, bones and brain [4]. In this case, the treatment involves immunotherapy, chemotherapy and targeted therapy, but these options are limited and ineffective in most cases [5]. Furthermore, side effects caused by the cytotoxicity of the chemotherapeutic agent considerably reduce the patient’s quality of life [6].

5-Fluorouracil (5FU) is an antimetabolite drug. The drug is an analogue of pyrimidine with hydrophilic characteristics [7]. It is the most extensively used antineoplastic agent in cancer chemotherapy given its broad-spectrum activity in solid tumors [7,8,9,10]. Thus, 5FU is the chemotherapeutic agent used for a variety of malignant tumors, including gastrointestinal, head and neck, and breast cancer [11] as well as tumors in the upper airways [12,13]. Additionally, 5FU significantly decreases the viability of melanoma cell lines [14,15].

However, nonspecific interactions of the drug with anionic biological molecules result in severe systemic toxicity [16,17]. Recent studies have dedicated efforts to increase the therapeutic efficacy of 5FU and reduce drug cytotoxicity by developing sustained release systems of this drug [17,18,19,20,21,22]. Nonetheless, some previous studies have reported severe side effects resulting from the systemic administration of 5FU [15] and low bioavailability after oral administration [19]. The cytosolic enzyme DPD (dihydropyrimidine dehydrogenase) metabolizing greater than 85% of the administered dose of 5FU in 5FUH2 (5,6-dihydro-5-fluorouracil), an inactive metabolite [11]. Its rapid metabolism leads to low drug exposure to tumor tissue, requiring repeated administration of high doses of the drug [23,24].

The pulmonary route can be an attractive alternative to offer protection against extensive degradation of 5FU given its low metabolic activity [25]. In addition, the pulmonary pathway has unique features such as large alveolar surface area, fine epithelium and extensive blood vasculature that allow systemic absorption and local drug action. Since the lung is organ most frequently affected by metastasis [4], this route of administration allows local action with high concentrated exposure of the drug directly at the target site [26]. All these factors lead to the possibility of reducing the dose of drug administered, which makes the lung administration extremely attractive to 5FU due to the severity of its adverse effects, besides favoring the therapeutic adherence because it is a noninvasive route.

However, the success of the treatment by this route depends essentially on two important requirements. First, the pharmaceutical formulation must have appropriate aerodynamic characteristics, allowing the particles to be conducted by airflow and deposited along the respiratory tract [27]. New methods of powder preparation have recently been described for various biomedical applications aiming to control the particle size, the morphological characteristics, as well as encapsulation efficiency [28,29,30]. The association of drugs with micro- and nanoparticle carrier systems has allowed to overcome the challenges in the development of inhaled therapeutic systems [27,31,32,33], and to modulate the drug release profiles [34,35]. Another important point to consider is the ability of the particles to circunvent the mechanisms of defense of the lung against foreign substances, besides being non-toxic and biocompatible (20). In this context, chondroitin sulfate, a sulfated glycosaminoglycan (GAG), is a natural, stable, biodegradable and low-toxicity polymer [36,37]. GAG is essential for tissue regeneration and for maintenance of cell functions [38], presenting great potential to reduce tissue damage and the inflammatory response in pulmonary tissues [39]. The negative charge allows this polymer to form complexes with positively charged molecules [40], such as 5FU, thus serving as a suitable structural candidate to formulate micro- and nanoparticles to carry this anti-tumor drug. In addition, increasing the exposure time of the drug in the lung tissue would offer a possible a reduction in the administered dose and an increase of the administration cycles, which can minimize some side effects. Additionally, the use of bioadhesive agents is also a strategy to enhance adsorption of the particles to the lung mucosa and promote the sustained release of the drug. Hydroxypropyl-methyl-cellulose (HPMC), a physiologically inert derivative of cellulose, is a potential mucoadhesive agent for pulmonary application [41,42].

Thus, the objective of this work was to develop novel formulations designed to treat advanced metastatic melanoma based on micro- and nanoparticles of 5FU for pulmonary delivery. Two innovative dry powder formulations containing 5FU were proposed. Conventional spray-drying (Mini Spray Dryer B-290, BÜCHI Labortechnik AG, Flawil, Switzerland) and piezoelectric atomization (Nano Spray Dryer B-90, BÜCHI Labortechnik AG) techniques were used to produce in a single step carrier systems of micro- and nanoparticles containing 5FU, respectively. The main contribution of this paper is that it considers a non-reported association of micro- and nanoparticles as a single therapeutic system for pulmonary delivery, with the aim of distributing drug the entire respiratory tract to reach deeper lung portions to treat melanoma.

## 2. Results

### 2.1. Dry Powders: Preparation and Physicochemical Characterization

The technology of the drying techniques used, coupled with the methodology designed for the development of carrier systems, proved to be successful in obtaining particles with different size ranges in a single step.

**Figure 1:**
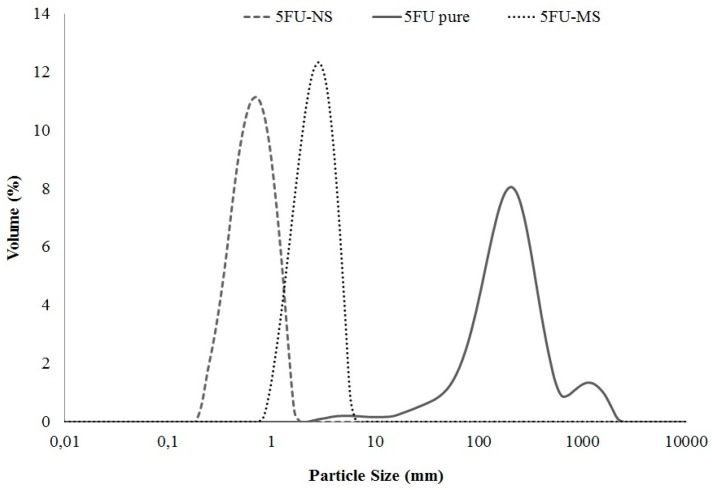
Particle size distribution obtained by laser diffraction (volume average analysis) for the 5FU nanoparticles obtained by piezoelectric atomization (5FU-NS) and 5FU microparticles of sustained release produced by conventional spray drying (5FU-MS) formulations, and drug 5FU pure (*n* = 3).

The average particle diameter and polydispersity index were determined, and both 5FU-NS and 5FU-MS systems presented unimodal and narrow particle size distribution range (span < 1.3), revealing homogeneity and reproducibility of the preparation method (Figure 1). The 5FU-NS formulation exhibited a large population of submicron particles with d_0.9_ of 1.057 ± 0.038 µm and D_[4,3]_ of 0.652 ± 0.033 µm. The 5FU-MS formulation exhibited a predominance of microparticles with d_0.9_ of 3.954 ± 0.111 µm and D_[4,3]_ of 2.546 ± 0.074 µm. The drug 5FU pure exhibited large particle sizes and multimodal profile with a wide particle size range (span > 2.9). The calculated theoretical aerodynamic diameters by tap density (d_ae_) were 0.322 ± 0.07 µm for 5FU-NS and 1.138 ± 0.54 µm for 5FU-MS.

The analysis of the drug content revealed that the concentration of 5FU is 23.34 ± 1.67% and 62.88 ± 1.77% of total solids in the 5FU-MS and 5FU-NS formulations, respectively. Both formulations exhibited efficient association of the drug to the particle with experimental drug content close to theoretical (approximately 60% (588.2 mg·g^−1^) and 20% (212.8 mg·g^−1^), respectively) and content uniformity based on the inter-day and intraday quantifications from three different batches.

### 2.2. Morphological Analysis

**Figure 2:**
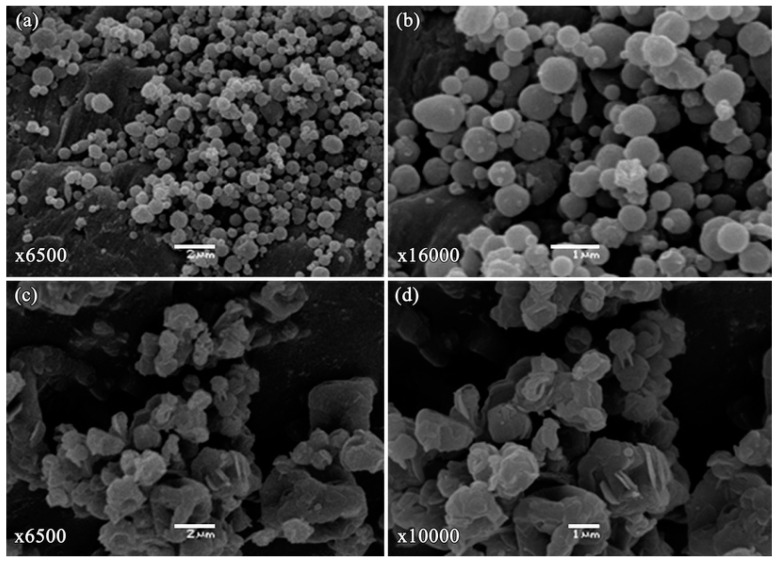
Scanning electron microscopy (SEM) images of dry powders: (**a**,**b**) 5FU-NS, obtained at 6500× and 16,000× magnifications, respectively; (**c**,**d**) 5FU-MS formulation, obtained at 6500× and 10,000× magnifications, respectively. Scale bars represent 2 µm (right) and 1 µm (left).

The images obtained by scanning electron microscopy (SEM) for 5FU-NS and 5FU-MS formulations are presented in Figure 2. Particles of the 5FU-NS formulation exhibited a spherical shape and smooth surface (Figure 2a), whereas the 5FU-MS formulation presented particles of irregular shape, the presence of concavities, and slightly roughened surface (Figure 2b). Neither of the formulations presented crystals, indicating no drug excess in the formulations, or pores in the surface of the particles.

### 2.3. Fourier Transformed Infrared Spectroscopy (FTIR)

**Figure 3:**
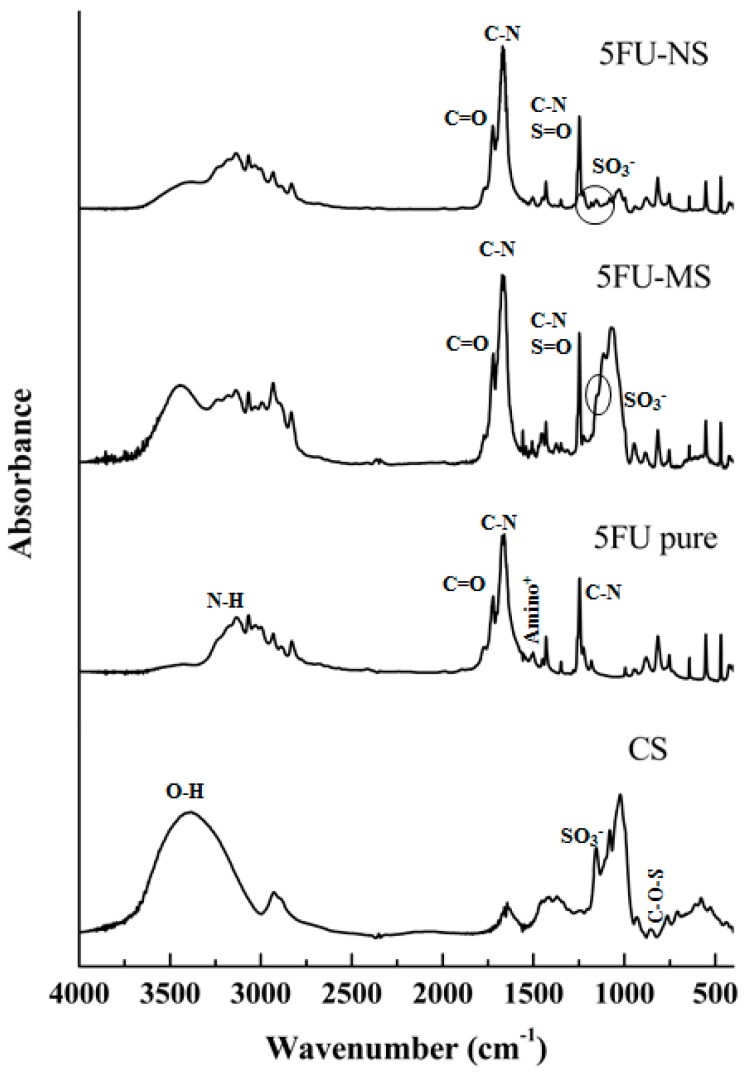
FTIR spectroscopy spectra of drug 5FU pure, chondroitin sulfate, and dry formulations, 5FU-MS and 5FU-NS.

The spectra obtained from each sample were collected and analyzed based on the infrared absorbance values of each wavelength detected, allowing verification of the characteristics of the compound formed by the drug and the polymer after the process of obtaining the particles compared with the chemical characteristics when pure (drug and polymer) (Figure 3).

The analysis of drug 5FU pure exhibited characteristic peaks at 1650.71 and 1246.02 cm^−1^ related to vibration of C–N bonds of the pyrimidine ring [22,43] and signals identified in 3136.25 and 1722.46 cm^−1^ were related to the stretching of the N–H and C=O bonds, respectively [43]. The band at 1550 cm^−1^ corresponds to the protonated amino group [44]. Regarding chondroitin sulfate, the spectral region above 2000 cm^−1^ corresponds to O–H stretching vibration, and the band identified in 1250 cm^−1^ is attributed to the S=O group. Characteristic bands of chondroitin sulfate were identified at 1636.64 cm^−1^. Bands generated by the amide I were noted at 855 cm^−1^, which resulted from the vibration of the C–O–S group, and at 1155.36 cm^−1^, which were attributed to the vibration of the SO_3_^−^ group [45].

In 5FU-NS formulation analysis, it was also possible to identify the same profile, demonstrating the presence of the drug and polymer in the formed system. From the spectra, it was also possible to observe a slight decrease in the intensity of the related signal to the vibration of SO_3_^−^ group, suggesting an interaction with the protonated amino group of the drug. In addition, a decrease in the band related to the O–H group stretching and detection of a signal at 3172.50 cm^−1^ were observed, suggesting the interaction between the drug and the polymer through hydrogen bonds. The same pattern was observed for the 5FU-MS formulation. However, due to the presence of HPMC coating the drug-chondroitin sulfate complex, the signal related to the vibration of the SO_3_^−^ group was obscured.

### 2.4. In Vitro Aerodynamic Performance

**Figure 4:**
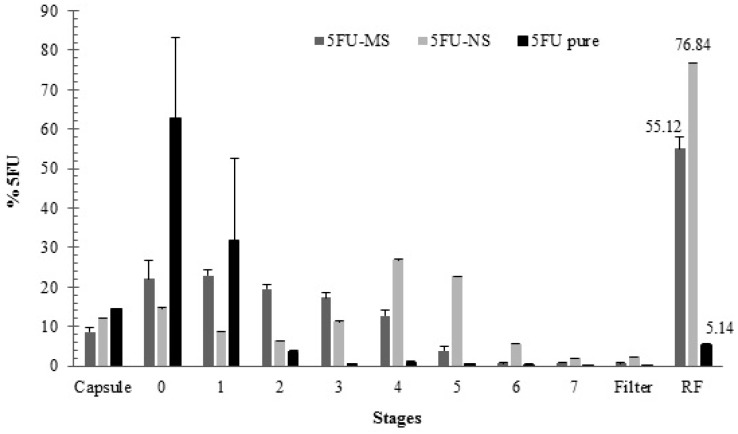
Deposition Performance (% 5FU) by ACI: 5FU-NS and 5FU-MS formulations, and drug 5FU pure (mean ± SD; *n* = 3).

In the aerodynamic performance analysis of the 5FU-NS and 5FU-MS formulations, it was possible to quantify the drug in all stages for both formulations, including stages 2–7, which represent the fraction of respirable particles (Figure 4 and Table 1).

The smaller particle size of 5FU-NS formulation was especially important regarding the performance, particularly considering the fraction of respirable particles (FR% = 76.84 ± 0.07%). The analysis exhibited significant deposition in stages 5–7 (Figure 4), predicting its efficient alveolar deposition [46]. On the other hand, the performance of the 5FU-MS formulation exhibited increased concentrations of entrapped drug in stages 2–5 (Figure 4) in relation to the total mass of powder delivered, providing medium to deep pulmonary deposition [47] and high fraction of respirable particles at 55.12 ± 2.98% (Table 1). The results obtained for the drug 5FU pure were significantly lower (*p* < 0.05) with an FR% of approximately 5% of the emitted dose (Table 1). All samples tested exhibited efficient powder dosage emission from hard gelatin capsule (ED%) (Table 1). The results obtained for the developed formulations 5FU-MS and 5FU-NS exhibited suitable dispersion characteristics for inhalation, allowing deposition throughout the pulmonary tissue (Figure 4). From the ACI, both formulations exhibited 50% of the total particle population less than 5 µm in diameter (Figure 4).

### 2.5. In Vitro Drug Release Assay

**Figure 5:**
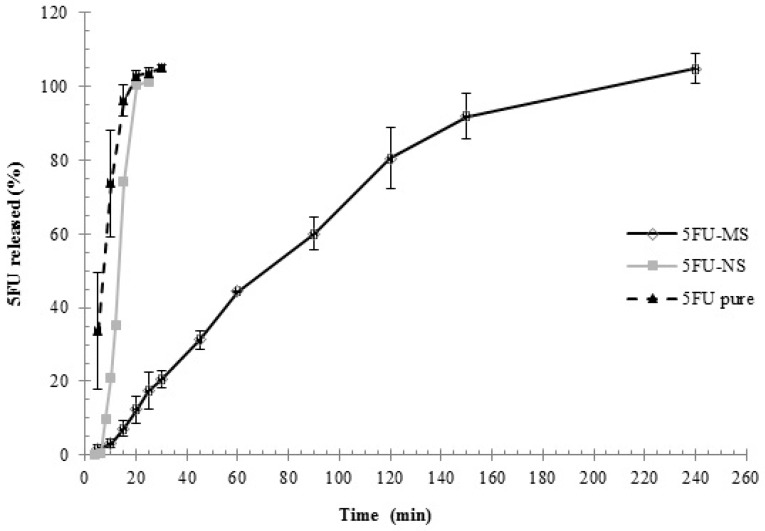
In vitro drug release profile: 5FU-NS and 5FU-MS formulations, and drug 5FU pure (mean ± SD; *n* = 3).

Release profiles of the 5FU-NS and 5FU-MS formulations and drug 5FU pure are shown in Figure 5. The result obtained for the control group exhibited an early rapid release of the drug compared with the 5FU-MS and 5FU-NS formulations (*p* < 0.05). In the first sampling time (5 min), a concentration greater than 30% of the total mass (33.67 ± 15.73%) was quantified. At 10 min, greater than 70% of the total concentration was available (73.72 ± 14.43%), indicating its rapid dissolution (Figure 5). The 5FU-NS formulation exhibited slower release of the drug when compared with the pure drug. At 5 min, less than 0.1% of the drug was available in the dissolution medium (0.08 ± 0.01%). At 10 min, only 20% of the total drug concentration was released (21.15 ± 12.07%). Then, 100% release was achieved at 25 min (Figure 5). In contrast, 5FU-MS formulation exhibited sustained and gradual drug release, resulting in 100% concentration of drug available in the medium after 4 h, which is significantly increased compared with the release time obtained for 5FU-NS and compared with control. The ability of the formulations to control and prolong the drug release was confirmed by mathematical modeling of these results. The model selection was based on the best correlation coefficient and MSC values obtained by fitting the experimental data. Mathematical modeling revealed that for 5FU-MS and 5FU-NS formulations, the best fit was observed by bi-exponential equation, which is characterized by two phases of release as demonstrated in the graph (Figure 5). The analysis of the release profile by Power Law model of the 5FU-MS (*n* = 0.76) and 5FU-NS (*n* = 1.21) formulations indicates that both have non-Fickian release mechanisms, anomalous transport type (0.43 < *n* < 0.89) and super-case II (*n* > 0.89), respectively.

### 2.6. Study of Mucoadhesive Properties In Vitro

#### 2.6.1. Mucoadhesive Performance

**Figure 6:**
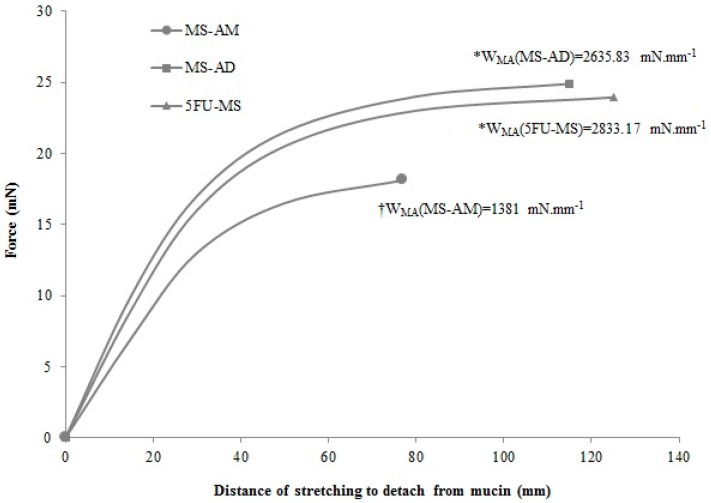
Mucoadhesive performance obtained for the 5FU-MS formulation, and MS-AM (5FU-MS formulation obtained in absence Methocel^TM^ F4M) and MS-AD (5FU-MS formulation obtained in absence of drug) powders, determined by the Force (mN) of the detachment from the mucin membrane versus Distance traveled (mm). The area under the curve represents the Mucoadhesion Work (*W*_MA_—mN·mm^−1^) (mean ± SD, *n* = 3; * No significant difference; † Significant difference of *).

The mucoadhesion work (*W*_MA_) performed to detach the powder formulations applied on the mucin membrane was calculated as the area under the curve obtained from the force of detachment (mN) versus the stretching distance to detach from mucin (mm) plot (Figure 6). The 5FU-MS formulation and MS-AD powder did not exhibit significant differences in the *W*_MA_ (*p* = 0.60), whereas both presented increased *W*_MA_ compared with the MS-AM formulation (*p* < 0.001).

Figure 6 demonstrates that the MS-AM powder needed reduced force and displacement to be detached from the mucin membrane, exhibiting a maximum peak force (*P*_MA_) at 18 mN and 77 mm, resulting in a *W*_MA_ = 1381 mN·mm^−1^. On the other hand, the 5FU-MS formulation exhibited the longest distance of detachment (124.98 mm) and one of the highest peak force (*P*_MA_ = 23.93 mN), which resulted in the highest value of *W*_MA_ (2833.17 mN·mm^−1^). For MS-AD powder, the peak force was *P*_MA_ = 24.88 mN at a detachment distance of 114.83 mm, resulting in the second highest value of *W*_MA_ (2635.83 mN·mm^−1^). Both 5FU-MS and MS-AD samples seem to maintain a constant force from the distance of 80 mm. From this point of the plot, these materials exhibit low additional force required to achieve longer distances until reaching the detachment point from the mucin membrane.

#### 2.6.2. Mucin Adsorption Capacity

**Figure 7:**
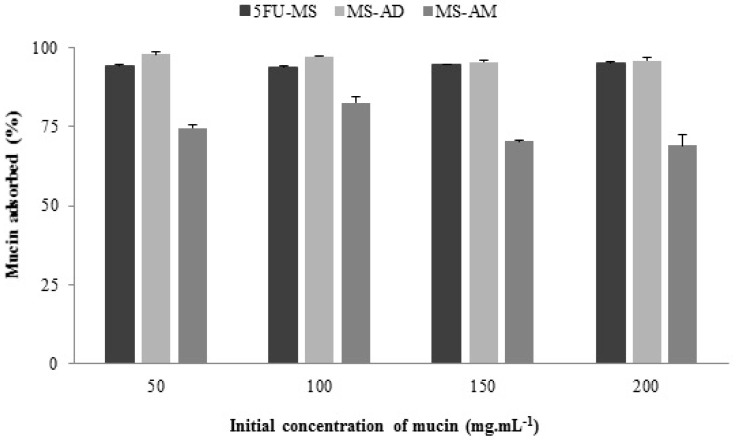
Concentration of mucin adsorbed (%) by 5FU-MS formulation, and MS-AM and MS-AD powders. Values obtained from the mean ± SD; *n* = 2).

The percentage of absorption of each formulation under different initial concentrations of mucin (µg mL^−1^) is presented in Figure 7. The high mucin absorption capacity of the 5FU-MS particles is noted with values close to 100% absorption for all concentrations tested. The same features are noted for the MS-AD powder, which exhibited no significant differences when compared with the 5FU-MS formulation (*p* > 0.05). However, in the absence of HPMC (MS-AM), the absorptive potential was significantly reduced, with maximum absorption values less than 85%. Furthermore, unlike 5FU-MS and MS-AD, the concentration of mucin absorbed (%) by the MS-AM decreases with increasing concentrations of mucin.

The data obtained on mucin adsorption capacity were analyzed using the Langmuir and Freundlich isotherm model. According to the linear correlation coefficient (*r*) obtained for the 5FU-MS formulation and the MS-AD and MS-AM powders, both isotherms represent the results appropriately. The parameters are presented in Table 2. Analysis of the Langmuir isotherm model verified that although a significant difference in the maximum capacity of mucin adsorption (*Q*_0_) (*p* < 0.05) was noted, the three samples exhibited efficient adsorption capacity (MS-AM > 5FU-MS > MS-AD). Another important aspect to consider is the energy absorption (*Q*_0b_), which was significantly increased for 5FU-MS (*Q*_0b_ = 200.0) compared with MS-AM powder (*Q*_0b_ = 2.17). The MS-AD sample exhibited negative energy absorption (*Q*_0b_ = −1428.57), indicating no chemical bonds with the mucin membrane. The absence of drug resulted in spontaneous and favorable adsorption (*RL* = 1) due to the presence of HPMC. According to the adsorption equilibrium parameter (*RL*) obtained in all the mucin concentrations tested, the 5FU-MS formulation was able to express different binding sites with mucin (*RL* = 1, favorable linear adsorption; values *RL* > 1 considered unfavorable to adsorption). The constants obtained using the Freundlich isotherm model corroborated those found using the Langmuir isotherm model. The 5FU-MS formulation exhibited significantly superior efficiency, as demonstrated by the adsorption capacity (K = 41.59) and favorable adsorption intensity at different sites (*n* = 3.33).

#### 2.6.3. Washability Test

**Figure 8:**
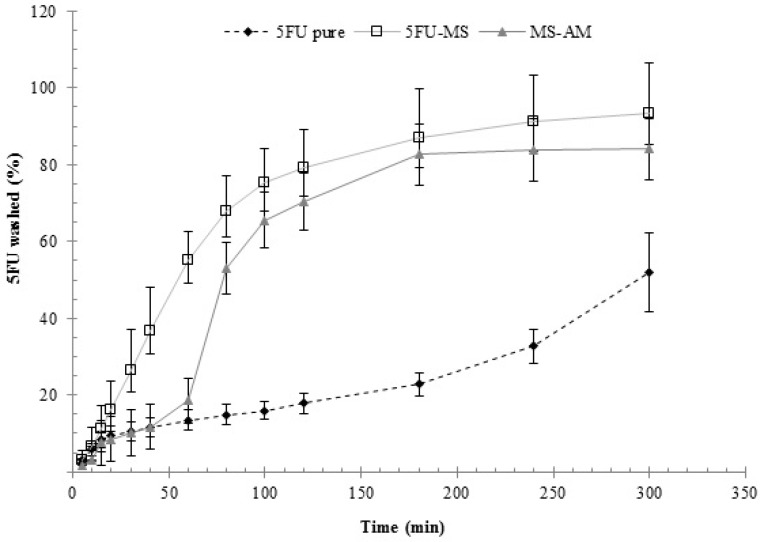
Washability profiles (0–300 min) of 5FU-MS formulation, MS-AM powder, and drug 5FU pure.

Washability profiles obtained for the 5FU-MS formulation, MS-AM powder, and the drug 5FU pure are presented in Figure 8. The results demonstrated that the drug concentration for MS-AM was gradually increased up to 60 min. At 80 min, an abrupt increase in the dissolved concentration is noted, and a stable state is observed at 180 min and beyond. In contrast, the 5FU-MS formulation exhibited gradual concentrations of the drug dissolved in the medium alongside the experiment, achieving a stable state only after 300 min. At 30 min and beyond, the 5FU-MS formulation exhibited significant differences in the dissolved drug concentrations (*p* < 0.05) in comparison with MS-AM powder and drug 5FU pure. Compared with drug 5FU pure, the difference is evident until the end of the experiment. At the end of the experiment, the receptor medium was quantified to determine the concentration of drug that permeated (% 5FU), resulting in values for the 5FU-MS and MS-AM formulation of 2.288 ± 0.668% and 1.794 ± 0.121%, respectively. The amount of drug 5FU pure was not quantifiable in the receptor medium.

### 2.7. Mitochondrial Activity Evaluation (MTT Assay)

**Figure 9:**
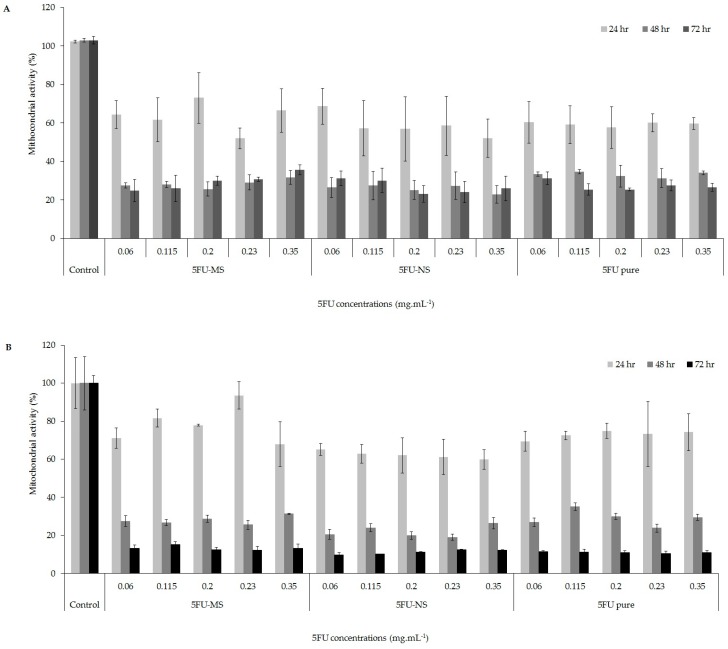
Cell viability (24, 48 and 72 h) of the 5FU-MS and 5FU-NS formulations at five concentrations. 5FU pure was used as a positive control. (**A**) A2058 cell viability; (**B**) A375 cell viability.

According to Figure 9 (Table S1, Supplementary Material), the formulations 5FU-NS and 5FU-MS were more effective against different melanoma tumor cell lines (A2058 and A375), significantly decreasing the cell viability in a time-dependent manner as well the drug 5FU pure in the same times. However, it can be seen that the effects of the formulations of micro- and nanoparticles, in both cell lines, were greater than the drug-free form (5FU pure), since similar results were observed for the percentage of mitochondrial activity with significantly lower concentrations (4.3 and 1.7 times lower than 5FU pure for 5FU-MS and 5FU-NS, respectively). This result evidences the effectiveness of the systems developed with respect to cytotoxic action in the tumor cell lines evaluated.

## 3. Discussions

The success in obtaining the carrier systems was evident according to the set of results presented. The selected spray techniques and the methodology designed to obtain micro- and nanoparticle carrier systems in this work proved to be efficient for the association of the 5FU drug. The compositions of the carrier systems and the selected preparation methods used in this paper have significantly impacted the characteristics of the particles, as confirmed by the morphological analysis and the average particle size. An important factor that contributed to the characteristics of each formulation was the drying technique employed. The 5FU-NS formulation was obtained by a recent drying technology, in which droplets of ultra-fine particle size are mildly dried, resulting in sub-micrometer solid particles [48,49], spherically shaped with smooth surface, as observed by SEM. On the other hand, the 5FU-MS formulation was obtained using conventional spray-drying technique, using mesh of 7 µm that results in the atomization of larger droplets. In this technique, the droplets are rapidly dried under high temperature and drying flow, resulting in the formation of larger particles. According to the images obtained by SEM, the particles of this formulation also presented surface visibly shriveled. These morphological characteristics of 5FU-MS particles occur due to the presence of HPMC in the formulation, which increases its viscosity. When subjected to rapid drying rates under high temperatures, the particles quickly generate a dry surface in atomized droplets. Then, the liquid contained inside is extracted by capillary action, generating high internal pressure. The high internal pressure subsequently leads to the collapse of the droplet, resulting in visible undulations on its surface [50,51,52,53].

HPMC is a hydrophilic matrix, and contact with aqueous medium promotes polymer chain relaxation and volume expansion [54], forming a film with rheological properties that allow controlled drug release [41,55]. HPMC gelation resulted in the formation of a diffusion barrier and a significant increase of drug release period from 5FU-MS compared with the 5FU-NS formulation. However, the complex formed between the drug and the chondroitin sulfate promoted the sustained release of the drug from the 5FU-NS formulation compared with 5FU pure.

The mathematical model applied to these results, which presented the coefficient value “*n*” estimated by the Power Law model, indicates that the anomalous release mechanism found for the 5FU-MS formulation occurs as a result of the combined and simultaneous action of solvent diffusion and swelling of the polymeric matrix processes (0.43 < *n* < 0.85), prolonging the time of drug release. On the other hand, the value of *n* > 0.85 observed for the 5FU-NS formulation suggests the super-case II release kinetics in which the solvent diffusion rate through the matrix is increased compared with the relaxation of the polymer (chondroitin), favoring the erosion process of the system [56]. The lower free fraction of drug in the medium is of great relevance given that its short half-life and toxic side effects are a significant disadvantage in the conventional form of administration. Thus, the association with chondroitin sulfate and the formation of a matrix system by incorporation of HPMC can effectively promote the presence of available drug for local or systemic action for a longer period. 

Aerodynamic characteristics of the developed formulations indicate that the combination of 5FU-MS and 5FU-NS as a single agent for pulmonary administration provides desirable physicochemical properties for drug release in the mid and deep lung regions (see Figure 1). Particle diameter is the primary factor for determining region deposition in the respiratory tract [47,57]. Powder formulations for inhalation produced with a high aerodynamic diameter suffer inertial impaction, depositing mainly in the oropharynx [27,31,58,59,60,61], and only very small particles (<1 µm) can reach the alveolar region [32], which is required for systemic action [26,33,57]. As previously shown, both formulations have adequate particle size distribution for efficient pulmonary deposition, including the population of very small particles constituting the 5FU-NS formulation. Hygroscopic agents associated with nanoparticles cause the retention of natural moisture from the lungs after inhalation, increasing the size and weight of the particles, ensuring lung retention [62,63] and preventing very small particles from exhalation due to the low inertia [64,65]. Chondroitin sulfate, a sulfated polysaccharide, is a member of a specific group of GAGs with swelling ability [66,67]. Mediated by the relative humidity of the respiratory tract, chondroitin sulfate present in the particles tends to prevent possible losses by exhalation and to ensure complete pulmonary deposition of ultrafine particles followed by gradual release of the drug.

According to the results of mucoadhesion essay, it is evident that the presence of HPMC resulted in increased *W*_MA_ by 5FU-MS, which was not altered in the absence of 5FU (MS-AD). Thus, the presence of HPMC in the 5FU-MS formulation ensures high adhesion potential to the pulmonary mucous membrane [68,69]. These results are consistent with the parameters obtained by the mucin adsorption test. In the absence of drug (MS-AD), a high adsorption capacity was noted. In the absence of HPMC (MS-AM), the adsorption energy was significantly reduced. Thus, the mucoadhesive capacity of the formulation is primarily conferred by the presence of HPMC, and the drug may positively influence this behavior. This influence is probably correlated with the opposite charges of 5FU and mucin, resulting in a second binding site.

The results presented also suggest the interaction of HPMC with the mucin membrane by physical adsorption, resulting in the entanglement of the chains by Van der Waals forces and hydrogen bonds [70,71], which is accentuated by the intimate contact between the bioadhesive system and the membrane due to the small particle size. Given the swelling capacity of HPMC, the system acquires mobility for interpenetrating the glycoprotein chains of the mucin, which is essential in the formation of these bonds. These data are confirmed by the constant “*n*”, revealing the heterogeneity of adsorption sites with favorable adsorption of mucin by the 5FU-MS system, as represented by Langmuir and Freundlich isotherms. A prolonged drug action in pulmonary administration requires a drug-containing system with good biomucoadhesion properties such that the formulation remains longer in the site of action. In this sense, the washability profile confirmed the mucoadhesive potential of the 5FU-MS formulation and possible resistance to pulmonary clearance, facilitating sustained release of the drug. This experiment also demonstrated the permeation ability of the 5FU-MS system, according to the total concentration of drug recovered in the receptor medium. Although the drug 5FU pure remained in contact with the mucous membrane for a longer time, it was not possible to quantify 5FU in the receptor medium. Additionally, the adhesion and permeation results obtained in the absence of HPMC due to the presence of chondroitin sulfate suggest a bioadhesive ability of 5FU-NS. This finding may indicate that the interaction between the 5FU-NS particles and the mucous membrane can overcome the mucus barrier for systemic action. These results may indicate the promising administration of the 5FU-MS system, prolonged local and systemic action of the drug, a reduction of the dosage and frequency of administration, and minimal toxic side effects, corroborating with the results obtained by the cellular viability analysis with both 5FU-MS and 5FU-NS formulations.

In the mitochondrial activity assay, it was possible to observe a high cytotoxic effect for both proposed formulations, in the five concentrations tested and in the evaluated times. The cytotoxic effect in the metastatic and non-metastatic melanoma cell lines showed the high efficacy of both formulations relative to drug 5FU pure, with concentrations of 1.7 and 4.3 times lower for 5FU-NS and 5FU-MS, respectively. This is of extreme relevance due to the serious side effects of toxicity. Thus, a high therapeutic effect can be achieved with lower administration doses. Other studies have also shown that the association of antitumor drugs to carrier systems such as doxorubicin, bromelain and imiquimod, are able to significantly decrease the viability of tumor cells (MCF-7 and SiHa) compared to the free drug [72,73,74]. This reinforces the potentiality of formulations developed with a view to increase the therapeutic efficacy of the 5FU drug, with lower doses.

## 4. Materials and Methods

### 4.1. Materials

Briefly, 5-fluorouracil (Nanjing Wellchem Enterprise, Nanjing, China), chondroitin sulfate (Summit Nutritionals International™, Lebanon, NJ, USA), sodium deoxycholate (Sigma-Aldrich, São Paulo, Brazil), hydroxypropyl-methyl-cellulose (Methocel™ F4M—Sigma-Aldrich, São Paulo, Brazil), mucin Type II (Mucin from porcine stomach, Sigma-Aldrich, São Paulo, Brazil), sulfuric acid (Vetec Química Fina Ltda, Rio de Janeiro, Brazil), and methanol (Merck, Rio de Janeiro, Brazil, high-performance liquid chromatographic-grade) were used in these studies. MTT reagent (3-(4,5-dimethylthiazolyl-2)-2,5-diphenyltetrazolium bromide) (Sigma Aldrich Inc., St. Louis, MO, USA). Epithelial human melanoma cell lines (A2058 (ATCC^®^ CRL-11147™) and A375 (ATCC^®^ CRL-1619™ All)), donated by prof. Dr. José Alexandre Barbuto—USP Institute of Biomedical Sciences, Tumor Immunology Laboratory (São Paulo, Brazil). All chemicals used were of pharmaceutical grade.

### 4.2. Production of Dry Powders for Lung Delivery

The formulations 5FU-MS and 5FU-NS were developed using the instruments Mini Spray Dryer B-290^®^ (BÜCHI Labortechnik AG) and Nano Spray Dryer B-90^®^ (BÜCHI Labortechnik AG), respectively, according to the compositions described in Table 3.

First, to obtain the 5FU-MS formulation, a dispersion of Methocel™ F4M (HPMC) was stored at 4 ± 1 °C overnight. Subsequently, a solution containing 0.05 g of chondroitin sulfate and 0.02 g of the surfactant sodium deoxycholate was prepared (A). Separately, a solution containing 0.1 g of 5FU in water (B) was also prepared. Solutions A and B were maintained under moderate stirring at 37 ± 1 °C for 50 min. After this period, solution (A) was poured into solution (B) with the aid of a funnel, stirred for 10 min, and then poured into the HPMC dispersion. The obtained suspension was spray-dried under the conditions described in Table 4.

To obtain the 5FU-NS formulation, solutions A and B were prepared as described above. Before mixing solutions A and B, 2 mL of acetone were added. At the end of the process, a translucent solution was obtained, which was fed into the Nano Spray Dryer B90^®^ linked to a dehumidifier unit to drying by vibrational atomization under the conditions described in Table 4.

The powders were collected from the collecting electrode with a brush in amber glass flasks that were hermetically sealed and stored in a dry place at room temperature. Each formulation was produced in triplicate, and the results are reported as the average of the batches. The yield of the drying process was calculated considering the total powder mass obtained and the initial solids mass present in the starting solution of each formulation (expressed in percentage).

### 4.3. Physicochemical Characterization of Dry Powders

All analyses were performed in triplicate (*n* = 3) from three independent batches produced for each formulation. Pure 5FU analyses were also performed.

#### 4.3.1. Mean Particle Size and Aerodynamic Diameter

The average particle diameter and size distribution were determined by laser diffraction using the Mastersizer^®^ 2000 equipped with a Scirocco dry disperser (Malvern Instruments, Worcestershire, UK). The 5FU refractive index was used (1.542), and the laser obscuration was 2%. It was also determined d_0.1_, d_0.5_, and d_0.9_ (corresponding to the diameters at 10, 50 and 90% cumulative volumes, respectively), D_[4,3]_ (weighted average) and Span (polydispersity index).

To determine the theoretical aerodynamic diameter (*d*_ae_), powder samples of 5FU-MS and 5FU-NS (3 g) were transferred to a graduated cylinder until 10 mL (apparent volume) was attained. The final volume of compression (v) was measured after 10, 500 and 1250 compaction using a roller Tapped Density Advisor (J. Engelsmann AG, Ludwigshafen, Germany). The test continued in series of 1250 impacts until a volume of less than 2% difference between two subsequent readings was achieved. The packing density (*d*) was calculated from the weighted mass obtained by the compressed volume (*d = m*/*v*, g/mL) according to the American Pharmacopoeia [75]. The aerodynamic diameter was calculated as described by Learoyd et al. (2008) [76] according to the equation $d_{\mathrm{ae}}=d\;\surd (\rho /\rho_{1})$, where *ρ* represents the density of compaction and *ρ*_1_ is equal to 1 g·cm^−3^. The value D_[4,3]_ obtained from laser diffraction was considered as the particle diameter (*d*).

#### 4.3.2. Drug Content

An analytical method was developed and validated to quantify the 5FU content and determine the uniformity of this drug in the powders by high-performance liquid chromatography with UV detection (HPLC-UV) according to the parameters specified in the ICH (1996) [77]. The method was linear for the quantification of pure drug 5FU. For the 5FU-NS formulation (*R²* = 0.999), the range was 30–105 µg·mL^−1^ (*R²* = 0.999), and the range was 5–140 µg·mL^−1^ for the 5FU-MS formulation (*R²* = 0.999). The method was precise, accurate and specific. Analyses were performed on a Perkin Elmer chromatograph (UV/VIS) using a C18 Phenomenex^®^ column (5 µm, 250 mm × 4.6 mm) and a pre-column from the same manufacturer. The mobile phase consisted of a mixture of ultrapure water and methanol (70:30, *v*/*v*) for the pure drug 5FU and 5FU-NS analysis and a solution of sulfuric acid HPLC grade (H_2_SO_4_ 0.005 M) for the 5FU-MS formulation. For the extraction process, the powders were dissolved in ultrapure water, sonicated (1 h-5FU-NS formulation and pure drug 5FU; 1 h 30 min-5FU-MS formulation), and filtered through a hydrophilic membrane (0.45 µm, Millipore^®^—Merck KGaA, Darmstadt, Germany). The chromatographic conditions used were as follows: isocratic flow rate of 0.8 mL·min^−1^, UV detection at 266 nm and 20 µL injection volume (total run: 5FU-NS/6 min; 5FU-MS/12 min). The 5FU content was expressed in mg·g^−1^ (milligrams of the drug per gram of powder). The 5FU content in the particles was calculated considering the concentration of recovered drug and the concentration added to the formulation (theoretical).

#### 4.3.3. Morphological Analysis

The shape and the particle surface were analyzed by scanning electron microscopy (SEM, JEOL Scanning Microscope JSM-5800, Tokyo, Japan) operated under high-vacuum condition (accelerating of 10 kV voltage). Each powder sample was placed on aluminum stubs covered with carbon tape, metallized with gold (Jeol Jee sputter 4B SVG-IN, Tokyo, Japan) and analyzed at different magnifications.

#### 4.3.4. Fourier Transformed Infrared Spectroscopy (FTIR)

FTIR analysis was performed in an infrared spectrophotometer IR Prestige-21 (Powder Transform Infrared Spectrophotometer, Shimadzu, Kyoto, Japan) to investigate the interaction of the substances in the formulations. Chondroitin sulfate, drug 5FU pure, and 5FU-NS and 5FU-MS powders were analyzed at room temperature within a range of 4000–400 cm^−1^ with a resolution of 4 cm^−1^, yielding 32 scans. The pellets were prepared in a hydraulic press under 5 tons force using KBr as background.

#### 4.3.5. In Vitro Aerodynamic Performance

The in vitro profile of pulmonary deposition of the powders was determined using the Dry Powder Inhaler Andersen Cascade Impactor (ACI-DPI, Apparatus D European Pharmacopoeia, Copley Scientific Limited, Erweka, Germany), consisting of 8 stages (0–7) with rotary impactor plates connected to a flow controller and vacuum pump. Samples of 5FU-MS (30 mg), 5FU-NS (20 mg), and the drug 5FU pure (21 mg) were transferred to hard gelatin capsules (size 3), which were introduced into the single-dose inhaler (Aerolizer^®^, Novartis, Basel, Switzerland) and perforated twice. This experiment was conducted under controlled inhalation rate of 28.3 L·min^−1^ for 4 s and a pressure of 4 kPa. After inhalation, the particles retained in each stage were rinsed off with ultrapure water. The concentration of 5FU was quantified by HPLC-UV according to the extraction method described in Section 4.3.2. The Fine Particle Dose (FPD), Fine Particle Fraction (FPF), Respirable Fraction (RF), and Dose Issued (DI) of inhaled powders for pulmonary absorption were determined according to the equations described by Meenach et al. (2013) [46].

#### 4.3.6. In Vitro Drug Release Studies

This experiment was adapted for the developed systems considering the specifications of ANVISA (RDC No. 31, August 2010) and USP (1999). Samples of 5FU-NS (26 mg), 5FU-MS (30 mg) formulations, and drug 5FU pure were transferred into hard gelatin capsules (size 3) surrounded by a steel belt. The dissolution medium was maintained in a water bath (36 ± 1 °C, 100 rpm) under sink conditions (150 mL of ultrapure water). Two-mL aliquots were collected and filtered through 0.45-µm Millipore^®^ membrane, and the drug concentration was determined by HPLC. Samples were collected at predetermined time intervals (5, 10, 15, 20, 25, 30, 45, 60, 90, 120, 150 and 240 min) replaced with an equal volume of fresh medium. The method was validated for 3 different batches of each formulation. The profile and mechanism of drug release from the particles were evaluated by mathematical modeling of the experimental data with the aid of Micromath Scientist^®^ software (version 3.0, Micromath^®^, Inc., Saint Louis, MO, USA). The release profiles were determined considering the suitability of the model by the criterion values selection (MSC), correlation coefficient (*r*) and best graphic setting. The release mechanisms were determined by Power Law model, which correlates exponentially the release of the drug and the time from polymeric systems, according to the equation *M_t_/M_∞_ = Kt^n^*.

### 4.4. Study of Mucoadhesive Properties In Vitro

#### 4.4.1. Mucoadhesive Performance

The mucoadhesive properties were measured on a texture analyzer (TA.XT Plus Texture Analyzer, Hamilton, MA, USA) using the Adhesive Test method (*n* = 6). The analysis was performed for the 5FU-MS formulation, and MS-AM and MS-AD were used as controls (composition described in Table 3).

First, mucin pellets were produced as the model membrane (11 mm diameter punch; 162 mg ± 2) and fixed to the stainless steel plate of the apparatus with the aid of moistened adhesive tape, which was maintained under heating at 37 °C. The tip of the probe was coated with adhesive tape (spherical end; 10 mm diameter), and a thin layer of powder was adhered to the tape. The mucin pellet was hydrated with 3 drops of ultrapure water at 37 °C, and excess liquid was removed with absorbing tissue after 1 min of contact. The test started with the probe at a height of 700 mm moved down at a speed of 2 mm·s^−1^ until contact with the mucin pellet by applying a minimum force (0.2 N-0.5 mm·s^−1^; during 300 s). The probe was returned to the initial stage at the same speed. The force required to separate the two surfaces was recorded to obtain a force versus time plot.

#### 4.4.2. Adsorption of Mucin

The ability to adsorb to mucin was performed for the 5FU-MS formulation (MS-AM and MS-AD analyzed as controls). Briefly, 20 mg of each sample formulation were dispersed in aqueous solutions of mucin prepared at concentrations of 50, 100, 150 and 200 µg·mL^−1^, and the solutions were vortexed and incubated at 37 °C for 1 h. Afterwards, the solutions were centrifuged (3000 rpm, 5 min). The supernatant was collected and subjected to protein quantification using the Lowry method to verify the amount of free mucin.

#### 4.4.3. Washability Assay

The test was performed in a modified Franz diffusion cell, presenting input and output channels from the wash solution compartment (ultrapure water at 37 °C), connected to a flow pump [69,78,79]. Porcine esophageal mucosa was used as the model membrane. To simulate the lung clearance mechanism, 10 mg of each sample were added to the membrane, and the wash flow started (0.2 mL·min^−1^). During the washing period, the cell was maintained in a water bath at 37 °C under moderate stirring for 420 min. Aliquots were collected from the output channel at predetermined times, filtered through 0.45 µm membrane filters and quantified by HPLC. At the end of the experiment, the concentration of permeated drug was determined by analysis of the liquid contained in the receiving environment.

### 4.5. Mitochondrial Activity Evaluation (MTT Assay)

The mitochondrial activity of two different epithelial human melanoma cell lines, A2058 (ATCC^®^ CRL-11147™, male melanoma from lymph node metastatic site) and A375 (ATCC^®^ CRL-1619™, female malignant melanoma) was evaluated by the MTT (3-(4,5-dimethylthiazol-2-yl)-2,5-diphenyltetrazolium bromide) assay as described by Amaral-Machado et al. (2016) [80], with some modifications. The assay was performed on three replicates for each cell line at five different concentrations (weight of powder/volume: 0.06, 0.115, 0.200, 0.230 and 0.350 mg·mL^−1^) for the 5FU-NS and 5FU-MS formulations, being that the drug concentration in the formulations represents 60 and 23% of the total solids, respectively. The concentrations were obtained by serial dilution of the loaded formulations directly in Roswell Park Institute medium (RPMI). The 5FU pure was directly dissolved in the RPMI at the same studied concentrations and the untreated cells were tested as a negative and positive control, respectively. Then 100 µL of A2058 and A375 cells in RPMI medium supplemented with 10% of fetal bovine serum were placed into a 96-well plates (7 × 10^4^ and 5 × 10^4^ cells/well, respectively) and incubated at 37 °C and 5% CO_2_ for a period of 24, 48, and 72 h in the presence of the aforementioned drug concentrations. Moreover, 100 µL of the MTT reagent at 1 mg·mL^−1^ was added to each well to analyze the mitochondrial activity by the MTT reduction in formazan crystals. After 4 h, formazan crystals were dissolved in 100 µL of ethanol and the absorbance was measured in a Multiskan Ascent Microplate Reader (Thermo Labsystems, Franklin, MA, USA) at 570 nm. The mitochondrial activity was evaluated by the relative absorbance value between the controls and the systems. Untreated cells were considered 100% of mitochondrial activity.

### 4.6. Statistical Analysis

Analysis of variance (ANOVA) followed by Tukey’s post hoc test for multiple comparisons was employed to analyze the experimental data. Differences between groups were considered significant at *p* < 0.05.

## 5. Conclusions

In this study, two different innovative pharmaceutical formulations consisting of inhalable powders containing 5FU and biocompatible and biodegradable excipients were successfully developed. Here, 5FU-MS was developed using HPMC and produced by conventional spray-drying technique, resulting in micrometric particles exhibiting prolonged drug release and attractive bioadhesive properties, suggesting lung mucoadhesive capacity. In addition, 5FU-NS was developed using the piezoelectric atomization technique, resulting in small-sized particles with a high fraction of respirable particles, indicating a potential ability for deposition in the deeper regions of the lung. Both formulations exhibited appropriate aerodynamic properties and dose uniformity for efficient pulmonary delivery. The formulations were tested for their cytotoxic action on melanoma cancer cells (A2058 and A375) and both presented a cytotoxic effect of 4.3 and 1.7 times greater than the drug 5FU pure. The results showed that the formulations 5FU-MS and 5FU-NS have favorable complementary properties for lung delivery. If combined in a unique therapeutic system, the powders presenting different granulometries, could be administered by means of a dry powder inhaler with a satisfactory drug distribution along the respiratory tract.

## Figures and Tables

**Table 1:** Aerodynamic properties by ACI.

	ED (%)	FPF (%)	RF (%)	DPF (µg of 5FU)
5FU-NS	92.02 ± 0.06	44.60 ± 0.24	76.84 ± 0.07	5201.73 ± 11.81
5FU-MS	94.14 ± 0.72	33.84 ± 0.58	55.12 ± 2.98	2198.40 ± 57.19
5FU pure	92.39 ± 4.46	2.78 ± 1.73	5.14 ± 0.55	591.97 ± 368.57

Values shown as mean ± SD (*n* = 3); Abbreviations: ED (emitted dose); FPF (fine particle fraction); RF (respirable fraction); FPD (fine particle dose).

**Table 2:** Mucin Adsorption by Langmuir and Freundlich Isotherms.

	sLangmuir Isotherm				Freundlich Isotherm		
	*Q*_0_	*Q*_0b_	*RL*	*r*	*K*	*n*	*r*
5FU-MS	100.00	200.00	1	0.994	41.59	3.33	0.531
MS-AD	83.33	*	1	0.931	26.30	1.77	0.990
MS-AM	142.86	2.17	>1	0.803	4.23	1.47	0.902

* Undefined result. Abbreviations: *r* = correlation coeficient, determines how well the model represents the data; *Q*_0_ = maximum adsorption capacity of mucin; *Q*_0b_ = energy adsorption; *RL* = equilibrium parameter (0 < *RL* < 1, favorable adsorption); *K* = Freundlich constant, represents the adsorption capacity; *n* = adsorption intensity constant (*n* = 1 energy sites are equivalente; *n* ≠ 1 distribution of energy sites varies with the adsorption capacity; 2 < *n* < 10 in favorable adsorption).

**Table 3:** Constitution of the formulations.

Component	5FU-MS *	5FU-NS *	MS-AD *	MS-AM *
5FU	0.1	0.1	-	0.1
Chondroitin Sulfate	0.05	0.05	0.05	0.05
Sodium Deoxycholate	0.02	0.02	0.02	0.02
Methocel F4M	0.3	-	0.3	-
H_2_O	100	98	100	100
Acetone	-	2	-	-

* The values shown represent the concentrations of solute (g) and solvent (mL). Abbreviations: 5FU-MS: formulation produced by Mini Spray Dryer B-290; 5FU-NS: formulation produced by Nano Spray Dryer B-90; MS-AD: powder produced by Mini Spray Dryer B-290 in the absence of drug; MS-AM: powder produced by Mini Spray Dryer B-290 in the absence of Methocel^TM^ F4M.

**Table 4:** Drying Parameters.

Mini Spray-Dryer B-290 ^1^		Nano Spray-Dryer B-90 ^1^	
Parameter ^2^	Condition *	Parameter ^2^	Condition *
Spray mesh (µm)	7	Spray mesh (µm)	4
Q-Flow	60	Airflow (L·min^−1^)	120
Inlet (°C)	130	Inlet (°C)	50
Outlet (°C)	70	Outlet (°C)	40
Aspirator (%)	100	CAP (°C)	62
Pump (%)	20	Mode *	Open
Nozzle Cleaner	1		
Mode *	Open		

^1^ Instrument coupled to accessory dehumidifier (Dehumidifier B-296); ^2^ According to the manufacturer’s manual; * Previously standardized.

## Supplementary Materials

The following are available online at http://www.mdpi.com/2079-4991/8/2/75/s1, Table S1. Mitochondrial activity evaluation of human melanoma cell lines against the 5-fluorouracil-loaded micro and nanoparticles at different concentrations and exposition times.

## References

[1] Melanoma Institute Australia 2017. https://www.melanoma.org.au/understanding-melanoma/stages-of-melanoma.

[2] Bhatia S., Tykodi S.S., Thompson J.A. (2009). Treatment of Metastatic Melanoma: An Overview. Oncology.

[3] Instituto Nacional de Câncer. http://www2.inca.gov.br/wps/wcm/connect/tiposdecancer/site/home/pele_melanoma/definicao+.

[4] Wolff K., Johnson R. (2009). Fitzpatrick’s Color Atlas and Synopsis of Clinical Dermatology.

[5] Yang S., Haluska F.G. (2004). Treatment of melanoma with 5-Fluorouracil or dacarbazine in vitro sensitizes cells to antigen-specific CTL Lysis through perforin/granzyme- and Fas-mediated pathways. J. Immunol..

[6] Garbe C., Peris K., Hauschild A., Saiag P., Middleton M., Spatz A., Grob J.-J., Malvehy J., Newton-Bishop J., Stratigos A. (2010). Diagnosis and treatment of melanoma: European consensus-based interdisciplinary guideline. Eur. J. Cancer.

[7] Singh B.N., Singh R.B., Singh J. (2005). Effects of ionization and penetration enhancers on the transdermal delivery of 5-fluorouracil through excised human stratum corneum. Int. J. Pharm..

[8] Wang X., Lin J., Zhang X., Liu Q., Xu Q., Tan R.X., Guo Z. (2003). 5-Fluorouracil-cisplatin adducts with potential antitumor activity. J. Inorg. Biochem..

[9] Gudasi K.B., Vadavi R.S., Shelke N.B., Sairam M., Aminahbavi T.M. (2006). Synthesis and characterization of novel polyorganophosphazenes substituted with 4-methoxybenzylamine and 4-methoxyphenethylamine for in vitro release of indomethacin and 5-fluorouracil. React. Funct. Polym..

[10] Rejinold N.S., Muthunarayanan M., Chennazhi K.P., Nair S.V., Jayakumar R. (2011). 5-fluorouracil loaded fibrinogen nanoparticles for cancer drug delivery applications. Int. J. Biol. Macromol..

[11] Malet-Martino M., Martino R. (2002). Clinical Studies of Three Oral Prodrugs of 5-Fluorouracil (Capecitabine, UFT, S-1): A Review. Oncologist.

[12] Grem J.L. (2000). 5-Fluorouracil: Forty-plus and still ticking. A review of its preclinical and clinical development. Investig. New Drugs.

[13] Longley D.B., Harkin D.P., Johnston P.G. (2003). 5-fluorouracil: Mechanisms of action and clinical strategies. Nat. Rev. Cancer.

[14] Senft D., Berking C., Graf S.A., Kammerbauer C., Ruzicka T., Besch R. (2012). Selective induction of cell death in melanoma cell lines through targeting of Mcl-1 and A1. PLoS ONE.

[15] Shenoy V.S., Gude R.P., Murthy R.S.R. (2013). In vitro anticancer evaluation of 5-fluorouracil lipid nanoparticles using B16F10 melanoma cell lines. Int. Nano Lett..

[16] Di Paolo A., Danesi R., Falcone A., Cionini L., Vannozzi F., Masi G. (2001). Relationship between 5-fluorouracil disposition, toxicity and dihydropyrimidine dehydrogenase activity in cancer patients. Ann. Oncol..

[17] Thomas A.M., Kapanen A.I., Hare J.I., Ramsay E., Edwards K., Karlsson G., Bally M.B. (2011). Development of a liposomal nanoparticle formulation of 5-fluorouracil for parenteral administration: Formulation design, pharmacokinetics and efficacy. J. Control. Release.

[18] Kaiser N., Kimpfler A., Massing U., Burger A.M., Fiebig H.H., Brandl M., Schubert R. (2003). 5-Fluorouracil in vesicular phospholipid gels for anticancer treatment: Entrapment and release properties. Int. J. Pharm..

[19] Lamprecht A., Yamamoto H., Takeuchi H., Kawashima Y. (2003). Microsphere design for the colonic delivery of 5-fluorouracil. J. Control. Release.

[20] Huang L., Sui W., Wang Y., Jiao Q. (2010). Preparation of chitosan/chondroitin sulfate complex microcapsules and application in controlled release of 5-fluorouracil. Carbohydr. Polym..

[21] Lu F., Lei L., Shen Y.Y., Hou J.W., Chen W.L., Li Y.G., Guo S.R. (2011). Effects of amphiphilic PCL–PEG–PCL copolymer addition on 5-fluorouracil release from biodegradable PCL films for stent application. Int. J. Pharm..

[22] Zhang C., Li G., Wang Y., Gui F., Zhang J., Huang Q. (2012). Preparation and characterization of 5-fluorouracil-loaded PLLA–PEG/PEG nanoparticles by a novel supercritical CO2 technique. Int. J. Pharm..

[23] Peters G.J., Lankelma J., Kok R.M., Noordhuis P., van Groeningen C.J., van der Wilt C.L., Meyer S., Pinedo H.M. (1993). Prolonged retention of high concentrations of 5-fluorouracil in human and murine tumors as compared with plasma. Cancer Chemother. Pharmacol..

[24] Tanaka F., Fukuse T., Wada H., Fukushima M. (2000). The history, mechanism and clinical use of oral 5-fluorouracil derivative chemotherapeutic agents. Curr. Pharm. Biotechnol..

[25] Shah N.D., Shah V.V., Chivate N.D. (2012). Pulmonary Drug Delivery: A Promising Approach. J. Appl. Pharm. Sci..

[26] Al-Qadi S., Grenha A., Carrion-Recio D., Seijo B., Remunan-Lopez C. (2012). Microencapsulated chitosan nanoparticles for pulmonary protein delivery: In vivo evaluation of insulin-loaded formulations. J. Control. Release.

[27] Beck-Broichsitter M., Schweiger C., Schmehl T., Gessler T., Seeger W., Kissel T. (2012). Characterization of novel spray-dried polymeric particles for controlled pulmonary drug delivery. J. Control. Release.

[28] Nangrejo M., Ahmad Z., Stride E., Edirisinghe M. (2008). Preparation of Polymeric and Ceramic Porous Capsules by a Novel Electrohydrodynamic Process. Pharm. Dev. Technol..

[29] Zhang C., Yao Z.-C., Ding Q., Choi J.J., Ahmad Z., Chang M.-W., Li J.-S. (2017). Tri-Needle Coaxial Electrospray Engineering of Magnetic Polymer Yolk−Shell Particles Possessing Dual-Imaging Modality, Multiagent Compartments, and Trigger Release Potential. ACS Appl. Mater. Interfaces.

[30] Raseck M., Ahmad Z., Cross R.B.M., Gil J.H., Wilton-Ely J.D.E.T., Miller P.W. (2017). Facile Preparation of Drug-Loaded Tristearin Encapsulated Superparamagnetic Iron Oxide Nanoparticles using Coaxial Electrospray Processing. Mol. Pharm..

[31] Ungaro F., d’Angelo I., Coletta C., d’Emmanuele D.V., Sorrentino R., Perfetto B., Tufano M.A., Miro A., La Rotonda M.I., Quaglia F. (2012). Dry powders based on PLGA nanoparticles for pulmonary delivery of antibiotics: Modulation of encapsulation efficiency, release rate and lung deposition pattern by hydrophilic polymers. J. Control. Release.

[32] Seville P.C., Li H., Learoyd T.P. (2007). Spray-Dried Powders for Pulmonary Drug Delivery. Crit. Rev. Ther. Drug Carr. Syst..

[33] Chow A.H., Tong H.H., Chattopadhyay P., Shekunov B.Y. (2007). Particle engineering for pulmonary drug delivery. Pharm. Res..

[34] Dutta R.C. (2007). Drug carriers in pharmaceutical design: Promises and progress. Curr. Pharm. Des..

[35] Baghirov H., Karaman D., Viitala T., Duchanoy A., Lou Y., Mamaeva V., Pryazhnikov E., Khiroug L., Davies C.L., Sahlgren C. (2016). Feasibility Study of the Permeability and Uptake of Mesoporous Silica Nanoparticles across the Blood-Brain Barrier. PLoS ONE.

[36] Volpi N., Maccari F. (2006). Electrophoretic approaches to the analysis of complex polysaccharides. J. Chromatogr. B Anal. Technol. Biomed..

[37] Lee E.S., Park K.H., Kang D., Park I.S., Min H.Y., Lee D.H., Kim S., Kim J.H., Na K. (2007). Protein complexed with chondroitin sulfate in poly (lactide-co-glycolide) microspheres. Biomaterials.

[38] Zou X.H., Jiang Y.Z., Zhang G.R., Jin H.M., Nguyen T.M., Ouyang H.W. (2009). Specific interactions between human fibroblasts and particular chondroitin sulfate molecules for wound healing. Acta Biomater..

[39] Uchida S., Itaka K., Chen Q., Osada K., Miyata K., Ishii T., Harada-Shiba M., Kataoka K. (2011). Combination of chondroitin sulfate and polyplex micelles from Poly(ethylene glycol)-poly{*N*′-[*N*-(2-aminoethyl)-2-aminoethyl]aspartamide} block copolymer for prolonged in vivo gene transfection with reduced toxicity. J. Control. Release.

[40] Zhang J.S., Imai T., Suenaga A., Otagiri M. (2002). Molecular-weight-dependent pharmacokinetics and cytotoxic properties of cisplatin complexes prepared with chondroitin sulfate A and C. Int. J. Pharm..

[41] De Mariscal P., Bell D.A., Roller S., Jones S.A. (1996). Fiber-based fat mimics methylcellulose gums. Handbook of Fat Replacers.

[42] Alpar H.O., Somavarapu S., Atuah K.N., Bramwell V.W. (2005). Biodegradable mucoadhesive particulates for nasal and pulmonary antigen and DNA delivery. Adv. Drug Deliv. Rev..

[43] Banerji B., Pramanik S.K., Mandal S., Maiti N.C., Chaudhuri K. (2012). Synthesis, characterization and cytotoxicity study of magnetic (Fe_3_O_4_) nanoparticles and their drug conjugate. RSC Adv..

[44] Lee S.-T., Mi F.-L., Shen Y.-J., Shyu S.-S. (2001). Equilibrium and kinetic studies of copper (II) ion uptake by chitosan-tripolyphosphate chelating resin. Polymer.

[45] Foot M., Mulholland M. (2005). Classification of chondroitin sulfate A, chondroitin sulfate C, glucosamine hydrochloride and glucosamine 6 sulfate using chemometric techniques. J. Pharm. Biomed. Anal..

[46] Meenach S.A., Anderson K.W., Hilt Z., McGarry R.C., Mansour H.M. (2013). Characterization and aerosol dispersion performance of advanced spray-dried chemotherapeutic PEGylated phospholipid particles for dry powder inhalation delivery in lung cancer. Eur. J. Pharm. Sci..

[47] Carvalho T.C., Peters J.I., Williams R.O. (2011). Influence of particle size on regional lung deposition—What evidence is there?. Int. J. Pharm..

[48] Li X., Anton N., Arpagaus C., Belleteix F., Vandamme T.F. (2010). Nanoparticles by spray drying using innovative new technology: The Buchi nano spray dryer B-90. J. Control. Release.

[49] Schafroth N., Arpagaus C., Jadhav U.Y., Makne S., Douroumis D. (2012). Nano and microparticle engineering of water insoluble drugs using a novel spray-drying process. Colloids Surf. B Biointerfaces.

[50] Foster T.P., Laetherman M.W. (1995). Powder characteristics of proteins spray-dried from different spray-dryers. Drug Dev. Ind. Pharm..

[51] Maa Y.F., Costantino H.R., Nguyen P.A., Hsu C.C. (1997). The effect of operating and formulation variables on the morphology of spray-dried protein particles. Pharm. Dev. Technol..

[52] Walton D.E., Mumford C.J. (1999). Spray dried productsð characterization of particle morphology. Trans. IChemE.

[53] Raffin R.P., Colome L.M., Haas S.E., Jornada D.S., Pohlmann A.R., Guterres S.S. (2007). Development of HPMC and Eudragit S100 blended microparticles containing sodium pantoprazole. Pharmazie.

[54] Brannon-Peppas L., Peppas N.A. (1990). Dynamic and equilibrium swelling behaviour of pH-sensitive hydrogels containing 2-hydroxyethyl methacrylate. Biomaterials.

[55] McCarron P.A., Donnelly R.F., Zawislak A., Woolfson A.D., Price J.H., Mcclelland R. (2005). Evaluation of a water-soluble bioadhesive patch for photodynamic therapy of vulval lesions. Int. J. Pharm..

[56] Bruschi M.L. (2015). Strategies to Modify the Drug Release from Pharmaceutical Systems.

[57] Newman S.P., Sutton D.J., Segarra R., Lamarca R., De Miquel G. (2009). Lung deposition of aclidinium bromide from Genuair, a multidose dry powder inhaler. Respiration.

[58] Chrystyn H. (1997). Is total particle dose more important than particle distribution?. Respir. Med..

[59] Brand P., Meyer T., Weuthen T., Timmer W., Berkel E., Wallenstein G., Scheuch G. (2007). Lung deposition of radiolabeled tiotropium in healthy subjects and patients with chronic obstructive pulmonary disease. J. Clin. Pharmacol..

[60] Kurmi B.D., Kayat J., Gajbhiye V., Tekade R.K., Jain N.K. (2010). Micro- and nanocarrier-mediated lung targeting. Expert Opin. Drug Deliv..

[61] Geller D.E., Weers J., Heuerding S. (2011). Development of an inhaled dry-powder formulation of tobramycin using PulmoSphere technology. J. Aerosol Med. Pulm. Drug Deliv..

[62] Longest P.W., Tian G., Li X., Son Y.J., Hindle M. (2012). Performance of combination drug and hygroscopic excipient submicrometer particles from a softmist inhaler in a characteristic model of the airways. Ann. Biomed. Eng..

[63] Son Y.J., Worth L.P., Hindle M. (2013). Aerosolization characteristics of dry powder inhaler formulations for the excipient enhanced growth (EEG) application: Effect of spray drying process conditions on aerosol performance. Int. J. Pharm..

[64] Jaques P.A., Kim C.S. (2000). Measurement of total lung deposition of inhaled ultrafine particles in healthy men and women. Inhal. Toxicol..

[65] Patton J.S., Byron P.R. (2007). Inhaling medicines: Delivering drugs to the body through the lungs. Nat. Rev. Drug Discov..

[66] Servaty R., Schiller J., Binder H., Arnold K. (2001). Hydration of polymeric components of cartilage—An infrared spectroscopic study on hyaluronic acid and chondroitin sulfate. Int. J. Biol. Macromol..

[67] Esko J.D., Selleck S.B. (2002). Order out of chaos: Assembly of ligand binding sites in heparan sulfate. Annu. Rev. Biochem..

[68] Sandri G., Rossi S., Ferrari F., Bonferoni M.C., Muzzarelli C., Caramella C. (2004). Assessment of chitosan derivatives as buccal and vaginal penetration enhancers. Eur. J. Pharm..

[69] Frank L.A., Sandri G., D’Autilia F., Contri R.V., Bonferoni M.C., Caramella C., Frank A.G., Pohlmann A.R., Guterres S.S. (2014). Chitosan gel containing polymeric nanocapsules: A new formulation for vaginal drug delivery. Int. J. Nanomed..

[70] Andrews G.P., Laverty T.P., Jones D.S. (2009). Mucoadhesive polymeric platforms for controlled drug delivery. Eur. J. Pharm. Biopharm..

[71] Carvalho F.C., Barbi M.S., Sarmento V.H., Chiavacci L.A., Netto F.M., Gremiao M.P. (2010). Surfactant systems for nasal zidovudine delivery: Structural, rheological and mucoadhesive properties. J. Pharm. Pharmacol..

[72] Antonow M.B., Asbahr A.C.C., Raddatz P., Beckenkamp A., Buffon A., Guterres S.S., Pohlmann A.R. (2017). Liquid formulation containing doxorubicin-loaded lipid-core nanocapsules: Cytotoxicity in human breast cancer cell line and in vitro uptake mechanism. Mater. Sci. Eng. C.

[73] Frank L.A., Chaves P.S., D’Amore C.M., Contri R.V., Frank A.G., Beck R.C.R., Pohlmann A.R., Buffon A., Guterres S.S. (2017). The use of chitosan as cationic coating or gel vehicle for polymeric nanocapsules: Increasing penetration and adhesion of imiquimod in vaginal tissue. Eur. J. Pharm. Biopharm..

[74] Oliveira C.P., Prado W.A., Lavayen V., Büttenbender S.L., Beckenkamp A., Martins B.S., Lüdtke D.S., Campo L.F., Rodembusch F.S., Buffon A. (2017). Bromelain-Functionalized Multiple-Wall Lipid-Core Nanocapsules: Formulation, Chemical Structure and Antiproliferative Effect Against Human Breast Cancer Cells (MCF-7). Pharm. Res..

[75] United States Pharmacopeial Convention (2006). United States Pharmacopeia (USP).

[76] Learoyd T.P., Burrows J.L., French E., Seville P.C. (2008). Chitosan-based spray-dried respirable powders for sustained delivery of terbutaline sulfate. Eur. J. Pharm. Biopharm..

[77] U.S. Department of Health and Human Services Food and Drug Administration (1996). Guidance for Industry—Validation of Analytical Procedures: Methodology.

[78] Bonferoni M.C., Rossi S., Ferrari F., Caramella C. (1999). A modified Franz diffusion cell for simultaneous assessment of drug release and washability of mucoadhesive gels. Pharm. Dev. Technol..

[79] Rossi S., Bonferoni M.C., Ferrari F., Caramella C. (1999). Drug release and washability of mucoadhesive gels based on sodium carboxymethylcellulose and polyacrylic acid. Pharm. Dev. Technol..

[80] Amaral-Machado L., Xavier-Júnior F.H., Rutckeviski R., Morais A.R.V., Alencar E.N., Dantas T.R.F., Cruz A.K.M., Genre J., Silva-Junior A.A., Pedrosa M.F.F. (2016). New Trends on Antineoplastic Therapy Research: Bullfrog (Rana catesbeiana Shaw) Oil Nanostructured Systems. Molecules.

