# Supplementary material for: An Inhalable Powder Formulation Based on Micro- and Nanoparticles Containing 5-Fluorouracil for the Treatment of Metastatic Melanoma
Source: Nanomaterials (Basel). 2018 Jan 30;8(2):75. doi: 10.3390/nano8020075 (PMC5853707; doi:10.3390/nano8020075)
Supplement: Supplementary file 1 [file nanomaterials-08-00075-s001.docx]

Table S1. Mitochondrial activity evaluation of human melanoma cell lines against the 5-fluorouracil-loaded micro and nanoparticles at different concentrations and exposition times.

| **Mitochondrial activity** | **Concentration (mg.mL^-1^)** | **Time (hours)** | **Mitochondrial activity (%) ± SD** | | |
| --- | --- | --- | --- | --- | --- |
|  |  |  | 5FU-MS | 5FU-NS | 5FU pure |
| **A2058 cellular line** | 0.06 | 24 | 64 ± 10 | 69 ± 13 | 60 ± 15 |
|  |  | 48 | 28 ± 2 | 26 ± 7 | 34 ± 2 |
|  |  | 72 | 25 ± 8 | 31 ± 5 | 31 ± 5 |
|  | 0.115 | 24 | 62 ± 16 | 57 ± 20 | 59 ± 14 |
|  |  | 48 | 28 ± 2 | 27 ± 10 | 35 ± 2 |
|  |  | 72 | 26 ± 10 | 30 ± 9 | 25 ± 4 |
|  | 0.2 | 24 | 62 ± 19 | 57 ± 24 | 58 ± 15 |
|  |  | 48 | 28 ± 5 | 25 ± 7 | 32 ± 8 |
|  |  | 72 | 26 ± 3 | 23 ± 6 | 25 ± 1 |
|  | 0.23 | 24 | 52 ± 7 | 59 ± 22 | 60 ± 7 |
|  |  | 48 | 29 ± 6 | 27 ± 10 | 31 ± 7 |
|  |  | 72 | 31 ± 2 | 24 ± 8 | 28 ± 4 |
|  | 0.35 | 24 | 67 ± 16 | 52 ± 14 | 60 ± 4 |
|  |  | 48 | 32 ± 5 | 23 ± 6 | 34 ± 1 |
|  |  | 72 | 36 ± 4 | 26 ± 9 | 27 ± 3 |
| **A375 cellular line** | 0.06 | 24 | 71 ± 5 | 65 ± 3 | 69 ± 5 |
|  |  | 48 | 28 ± 3 | 21 ± 3 | 27 ± 2 |
|  |  | 72 | 13 ± 2 | 10 ± 1 | 12 ± 1 |
|  | 0.115 | 24 | 82 ± 5 | 63 ± 5 | 72 ± 2 |
|  |  | 48 | 27 ± 2 | 24 ± 2 | 35 ± 2 |
|  |  | 72 | 15 ± 1 | 10 ± 1 | 11 ± 1 |
|  | 0.2 | 24 | 78 ± 1 | 62 ± 9 | 75 ± 4 |
|  |  | 48 | 29 ± 2 | 20 ± 2 | 30 ± 2 |
|  |  | 72 | 12 ± 1 | 11 ± 1 | 11 ± 1 |
|  | 0.23 | 24 | 94 ± 7 | 61 ± 9 | 73 ± 17 |
|  |  | 48 | 26 ± 2 | 19 ± 2 | 24 ± 2 |
|  |  | 72 | 12 ± 2 | 13 ± 1 | 11 ± 1 |
|  | 0.35 | 24 | 68 ± 12 | 60 ± 5 | 74 ± 10 |
|  |  | 48 | 31 ± 1 | 26 ± 3 | 29 ± 2 |
|  |  | 72 | 13 ± 2 | 12 ± 1 | 11 ± 1 |

**Abbreviations:** 5FU-MS: formulation produced by Mini Spray Dryer B-290; 5FU-NS: formulation produced by Nano Spray Dryer B-90; 5FU pure: free drug. SD: Standard deviation.
